# A Review on Plant Cellulose Nanofibre-Based Aerogels for Biomedical Applications

**DOI:** 10.3390/polym12081759

**Published:** 2020-08-06

**Authors:** H.P.S. Abdul Khalil, A.S. Adnan, Esam Bashir Yahya, N.G. Olaiya, Safrida Safrida, Md. Sohrab Hossain, Venugopal Balakrishnan, Deepu A. Gopakumar, C.K. Abdullah, A.A. Oyekanmi, Daniel Pasquini

**Affiliations:** 1 School of Industrial Technology, Universiti Sains Malaysia, Penang 11800, Malaysia; essam912013@gmail.com (E.B.Y.); sohrab@usm.my (M.S.H.); deepu1789@gmail.com (D.A.G.); ck_abdullah@usm.my (C.K.A.); abdulkan2000@yahoo.com (A.A.O.); 2 Management Science University Medical Centre, University Drive, Off Persiaran Olahraga, Section 13, Shah Alam Selangor 40100, Malaysia; 3 Department of Industrial and Production Engineering, Federal University of Technology, Akure 340271, Nigeria; ngolaiya@futa.edu.ng; 4 Department of Biology Education, Faculty of Teacher Training and Education, Universitas Syiah Kuala, Banda Aceh 23111, Indonesia; saf_rida@unsyiah.ac.id; 5 Institute for Research in Molecular Medicine, Universiti Sains Malaysia, Penang 11800, Malaysia; venugopal@usm.my; 6 Chemistry Institute, Federal University of Uberlandia-UFU, Campus Santa Monica-Bloco1D-CP 593, Uberlandia 38400-902, Brazil; danielpasquini2013@gmail.com

**Keywords:** cellulose, nanofibre, aerogel, sustainable, biomedical applications

## Abstract

Cellulose nanomaterials from plant fibre provide various potential applications (i.e., biomedical, automotive, packaging, etc.). The biomedical application of nanocellulose isolated from plant fibre, which is a carbohydrate-based source, is very viable in the 21st century. The essential characteristics of plant fibre-based nanocellulose, which include its molecular, tensile and mechanical properties, as well as its biodegradability potential, have been widely explored for functional materials in the preparation of aerogel. Plant cellulose nano fibre (CNF)-based aerogels are novel functional materials that have attracted remarkable interest. In recent years, CNF aerogel has been extensively used in the biomedical field due to its biocompatibility, renewability and biodegradability. The effective surface area of CNFs influences broad applications in biological and medical studies such as sustainable antibiotic delivery for wound healing, the preparation of scaffolds for tissue cultures, the development of drug delivery systems, biosensing and an antimicrobial film for wound healing. Many researchers have a growing interest in using CNF-based aerogels in the mentioned applications. The application of cellulose-based materials is widely reported in the literature. However, only a few studies discuss the potential of cellulose nanofibre aerogel in detail. The potential applications of CNF aerogel include composites, organic–inorganic hybrids, gels, foams, aerogels/xerogels, coatings and nano-paper, bioactive and wound dressing materials and bioconversion. The potential applications of CNF have rarely been a subject of extensive review. Thus, extensive studies to develop materials with cheaper and better properties, high prospects and effectiveness for many applications are the focus of the present work. The present review focuses on the evolution of aerogels via characterisation studies on the isolation of CNF-based aerogels. The study concludes with a description of the potential and challenges of developing sustainable materials for biomedical applications.

## 1. Introduction

Cellulose fibres derived from plant fibre have been widely used as reinforcements in polymers for packaging and biomedical applications. The use of aerogel in the biomedical application is on the increase, especially in tissue repair. The reparation of aerogel is difficult, time consuming and a major challenge in this research phase. However, extensive studies to develop materials of cheaper and better properties with high prospects and effectiveness for many applications are the focus of the present work. Aerogel contains more than 99% air and is a lightweight material, often prepared and manufactured in multi-shape structures depending on the required application. It has been manufactured from many inorganic and organic sources [1,2].

More recently, the development of nanobiotechnology aerogels has been possible from a variety of nanomaterials such as chitosan nanoparticles, cellulose nano fibres (CNFs), metals like silver and alginate, etc. Many scientists have worked on studies to develop aerogel with cheaper and better properties for several applications [3]. The classification of aerogels is based on the nature of the materials used for the preparation. Some examples are mesoporous silica aerogels, graphene-based aerogels, composite aerogels, and aerogel catalysts. Previous studies reported that hydroxyapatite silica aerogel from amorphous silica rice husk served as a biomaterial for in vitro biocompatibility as an alternative biomaterial for biomedical applications. The sol–gel ambient pressure drying method was used for the fabrication of silica aerogel; the results obtained from the characterisation studies revealed that the synthesised aerogel was effective for the nucleation of apatite (Sani et al., 2017). Due to its high electrical conductivity, ultra-lightweight, large specific surface area graphene aerogel has been applied for temperature and pressure sensing, as well for material elasticity (Mao et al., 2020). Silica aerogel–polyvinyl alcohol composite aerogel was synthesised at ambient pressure for drug delivery. Bioaerogels made from polysaccharides have received remarkable attention (Soorbaghi et al., 2019). They have been used widely in medical applications such as in tissue engineering and drug delivery.

The majority of nanomaterials used in the manufacturing of aerogels are biopolymers. Biopolymers have been preferably used in biomedical and environmental applications due to their abundance, renewable source, low toxicity, biocompatibility and biodegradability. Various plant and animal biopolymers have been used in the preparation of aerogels. This includes cellulose, collagen, chitosan, alginate, etc. However, plant biopolymers are preferred due to their availability, biocompatibility, biodegradability and renewability potential. Cellulose has been reported as the most abundant natural polymer, and is mostly used in the preparation of aerogels more than other biopolymers. Cellulose is mainly derived from biomass, and it is the main composite of the plant cell walls [4]. It has also been widely isolated from other living organisms such as bacteria [4]. Bacterial cellulose does not contain impurities such as wax, lignin, pectin, and hemicelluloses, which were commonly present in cellulose derived from plants [4]. Plant cellulose can be isolated from different plants, parts of a plant or plant waste. According to the literature, the most used plants are soybean hulls, wheat straw, sugarcane, rice straw and husks, palm oil residue, pineapple, banana leaf fibre, bagasse, hemp and flax straws [5,6,7].

Thin cellulose fibres, often called cellulose nanofibres (CNFs), have a high crystallinity, great mechanical stiffness and strength [8]. Their diameter is approximately 3 nm, and their micron-scale lengths have both crystalline and amorphous sections. CNF can be isolated from the microfibres of plant cellulose [9]. It is often prepared by the mechanical fibrillation of cellulose and its yield depends on the source of the cellulose and the preparation method. The obtained CNF can include the ultrafine grinding of cellulose biomass or homogenisation and microfluidisation [10]. In some cases, chemical, enzymatic or even mechanical pre-treatment of CNF is done to enhance the quality of materials, reduce the energy input or achieve other purposes [11]. Owing to the unique properties of CNF and its ability to be incorporated with different materials, various potential applications have been suggested, tested and achieved.

**Figure 1:**
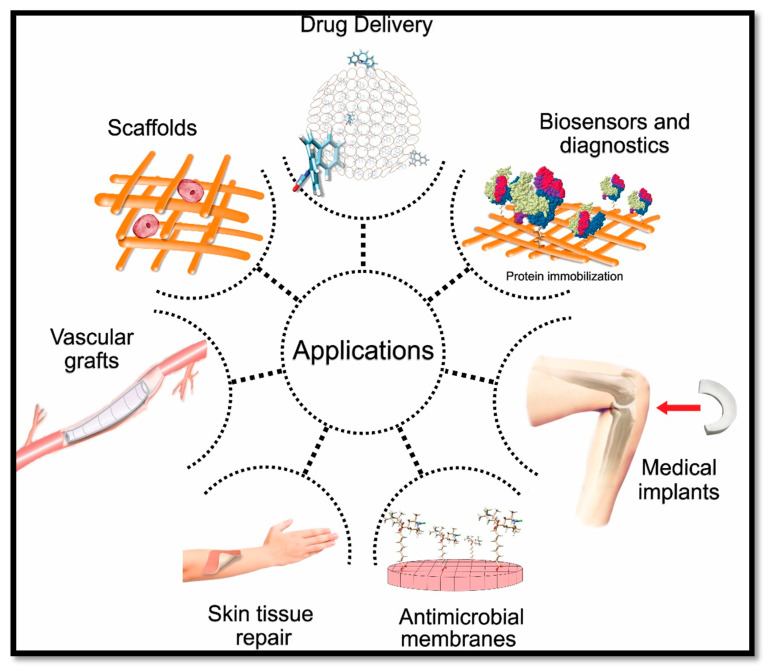
Cellulose nanofibre (CNF)-based aerogel in biomedical applications.

The applications of CNF-based materials include composites, organic–inorganic hybrids, the formation of gels, foams, aerogels/xerogels, coatings and nano-paper. Those materials have been widely studied and experimented in many medical and biomedical applications. Biomedical applications of CNF include drug delivery, scaffold fabrication, biosensing and diagnostic purposes, antimicrobial wound dressing, medical implants and vascular grafts, as shown in Figure 1.

**Figure 2:**
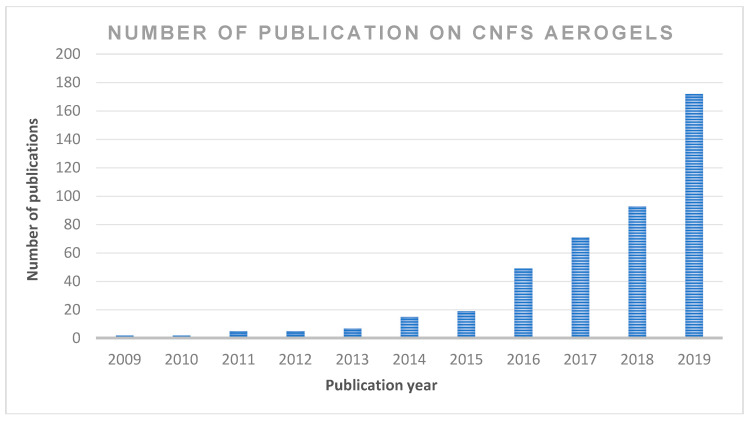
The number of scientific publications contributing to the subject “cellulose nanofibre; CNF and aerogel” by year (search done through ScienceDirect on 6 June 2020 (from 2009 to 2019).

The application of cellulose-based materials is widely reported in the literature. However, only a few studies discuss the potential of cellulose nanofibre in detail, owing to the fact that these properties, which include CNF applications in composites, organic–inorganic hybrids, gels, foams, aerogels/xerogels, coatings, nano-paper production, bioactive and wound dressing materials, bioconversion and other future potential applications, have not been extensively reviewed. Moreover, CNF use in the production of aerogels has a wide application in many areas. However, the use of sustainable aerogel in biomedical applications has not been discussed in detail. Publications on cellulose nanofibre aerogel used in biomedical applications continue to attract the interest of readers; however, there has not been an updated review on this subject. Figure 2 summarises the number of publications in the past 10 years in the ScienceDirect database using cellulose nanofibre. The focus of the present review is on the recent advancements in the preparations, properties, chronological studies and stages of development of aerogels for biomedical applications. This review includes recent trends regarding the application of cellulose nanofibre aerogels, as well as the prospects and challenges of the potential applications, especially in sustainable biomedical applications.

## 2. Chronological Studies

The cloudy appearance of aerogel has given it various nicknames such as frozen or blue smoke. Several studies have been conducted over the decades using different materials based on the required properties [12,13]. A significant development in the ease of aerogel preparation has been achieved over the years, though there is still much to be done. In recent studies, different methods have been explored to produce aerogel with organic and inorganic materials. The demand for aerogel for biomedical applications has been on the rise due to an increase in the rate of population and industrialisation. There are several requirements for materials used to produce aerogel and one of the critical factors reported have been their nontoxicity potential. Two critical factors mainly influenced the evolution of aerogel: precursor materials and preparation methods. Novel materials and techniques have been developed over the decades. Recently, many micro and nano-sized polymers have been extracted and used in aerogel fabrication. Polymers blends, composites, stabilising agents, and reinforced fibre particles have all been well studied and tested for many potential applications. The chronological order of aerogel evolution is presented in Table 1. Aerogel was discovered in 1931 by Samuel Stephens Kistler by an experiment in Stockton, California, USA. The result of his findings was reported as the first landmark invention. Kistler’s first aerogel was prepared by sodium silicate (water glass) and a silica gel was formed by heating in an autoclave at a temperature above the critical temperature and pressure of the gel material. This approach, after a while, became known as supercritical drying. Kistler describes the aerogel (air + gel) as a gel system in which the liquid has been replaced with a gas network of solid particles with only a slight shrinkage of the gel observed [3].

However, after the successful preparation of the silica aerogel, Kistler synthesised many aerogels with organic metal oxides, including polymers [14,15]. Moreover, Kistler developed and patented a new approach in the preparation of silica aerogel by using trichloro-methyl silane for the silylation to produce water-repellent aerogels, which are known as hydrophobic silica aerogels [16]. In 1968, Kistler’s route to obtain water glass was developed by Teichner and Nicoloan. They prepared the first sol–gel chemistry silica aerogel. Their method aimed to replace the water glass with tetramethyl orthosilicate (TMOS), which was easier to remove during supercritical drying.

Consequently, experiments on and applications of aerogel were reported for the first time in 1974. This effort resulted in the development of a Cherenkov radiation detector and this led to the start of the mass production of aerogel. In 1989, the sol–gel approach was developed. In the process, many aerogels with extremely low density and high porosity were produced [17].

Many advances have been reported in the literature regarding aerogel development, synthesis, and physical-chemical properties. It was observed that supercritical heating was time consuming. To proffer a solution to this challenge, John Poco, in 1996, developed a route known as supercritical extraction (RSCE) to speed up the production process [20]. X-aerogels were developed a year later by Leventis et al. by cross-linking the structures of silica with di-isocyanates, resulting in an ultralight aerogel with extreme physical and mechanical properties [33]. A nanoporous metal aerogel was developed more recently with metals to obtain a special type of aerogel with extremely high porosity and surface area and an ultra-low density [34]. A completely metal aerogel was prepared and is known as an iron oxide xerogel, as it is composed mainly of iron [24]. NASA reported the development of a polymerised aerogel known as polyimide aerogel. The polyimide aerogel was obtained by cross-linking metals in modified conditions and the invention was 500 times more mechanically strong than traditional silica aerogel [35,36]. The chronological order analysis showed the diversity of precursor materials and composites that have been integrated into aerogel fabrication

The methods of aerogel preparation started with the supercritical drying technique, converting the liquid part of the mixture into a gas within the silica nanopores, without the structure collapsing. Although supercritical drying for aerogel preparation was effective, it was time consuming, significantly expensive, and the internal structure of the aerogel could not be designed [37]. Supercritical drying, at present, has been modified by replacing the previously used alcohol with CO_2_ but with little improvement in terms of the disadvantages of the method. The sol–gel route is another technique that has been used by replacing water glass with tetramethyl orthosilicate (TMOS), then removing it at supercritical conditions. Other modified techniques such as freeze-casting, foaming, freeze-drying and ambient pressure drying techniques improved on the previous methods.

At present, the ease of aerogel fabrication has been significantly improved by advanced methods adapted from rapid prototyping. Stereolithography is one of the recent techniques that has been developed for the fabrication of three-dimensional aerogel [38]. Stereolithography is one of the rapid prototyping technologies, which depends on computer-aided design for the fabrication process. Different shapes of aerogel scaffolds have been prepared recently using this method [39,40,41]. Rapid prototyping is a series of computer-based techniques that have revolutionised the fabrication of aerogels in the past two decades with the precise control of the complex 3D macrostructure and microstructure. This method allowed for the fabrication of desirable shapes of aerogels and facilitated the preparation process [42].

Furthermore, the direct 3D printing of aerogel has enhanced the possibility of controlled aerogel pore sizes and their 3D internal structural network. A few research works have been carried out on the fabrication of nanowire aerogels from novel materials [43,44,45]. The advent of this novel technique is expected to eliminate complex preparation methods such as gelatinisation, organic solvent replacement, retrogradation and supercritical CO_2_ drying in the future [46]. Furthermore, a variety of precursor materials now exist that possess different properties, giving researchers the chance to develop novel aerogels for different applications in many areas and enhance their durability. Novel aerogels have been developed for water absorbance, including cellulose-based aerogels for oil spills, aerogels with thermal superinsulation and aerogels for biomedical applications.

## 3. Preparation of CNF Aerogel and Properties

### 3.1. Isolation of Plant Cellulose Nanofibres (CNFs)

**Figure 3:**
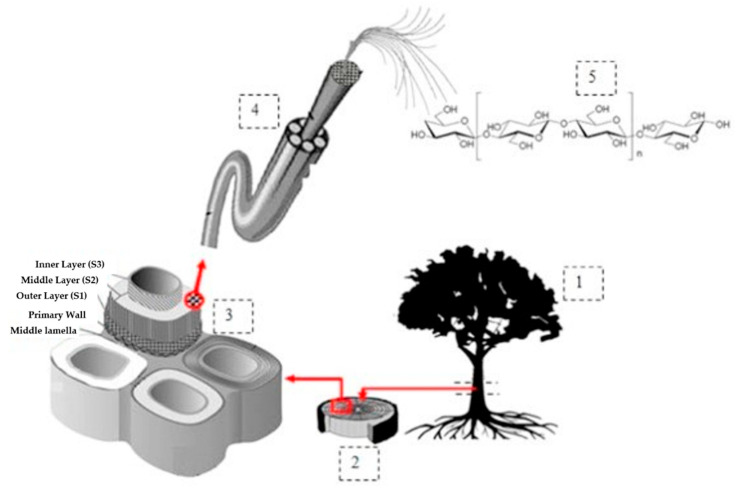
Schematic drawing of plant cellulosic fibre hierarchical structure. (**1**) Bark cambium pith, (**2**) cambium (**3**) mature cell wall, (**4**) cellulose fibre, and (**5**) chemical structure of cellulose (cited from H.P.S. Abdul Khalil et al. [50]). Copyright 2016. Reused with permission from Elsevier Ltd.

Over the years, cellulose nanofibre (CNF)-based aerogels have been widely and intensively studied and applied in many biomedical fields [47,48]. This is due to their attractive and unique characteristics, such as their ultra-light weight (99.98% air by volume), high porosity and excellent strength. CNFs have promising potential in various medical applications due to their large surface area, high tensile strength and low coefficient of thermal conductivity. Cellulose, which is the precursor material in the production of CNF, is present in all plant cell walls. Cellulose is present in algae, some fungi, marine organisms of the tunicate family, invertebrates, and some Gram-negative bacteria [49]. Plant fibres are found in relative abundance and are renewable. A plant cell wall comprises primary and secondary cell walls. The primary cell wall has a thin outer layer, and the secondary wall is composed of three layers (Figure 3).

**Figure 4:**
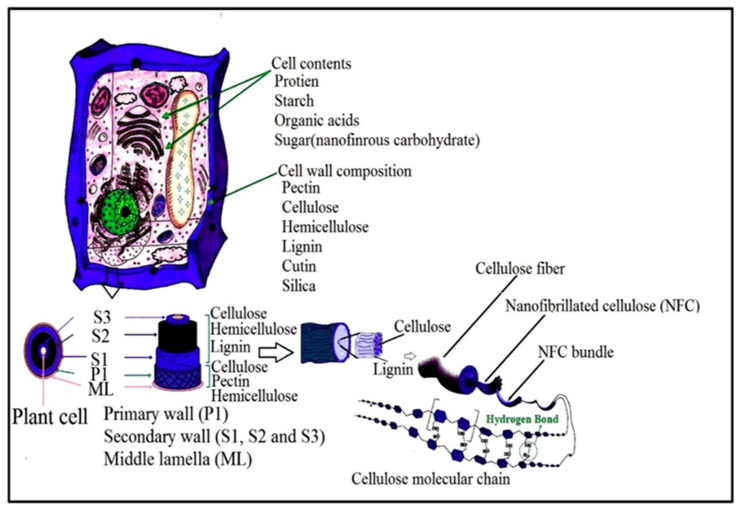
Illustration of plant cell wall and structure of native plant cellulosic fibre (adapted from *Journal of Saudi Chemical Society*; Mishra [54]. Copyright 2018. Reused with permission from Elsevier Ltd.

**Figure 5:**
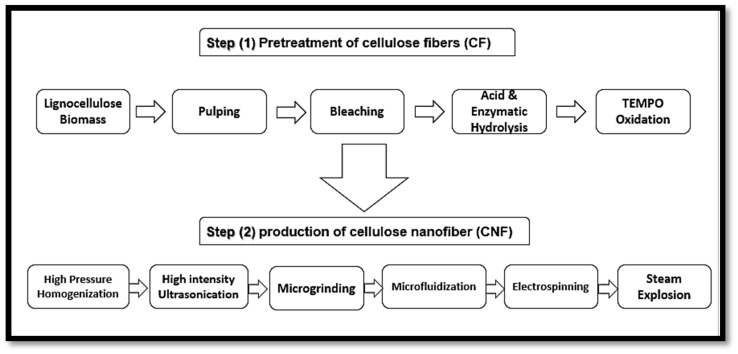
Steps of plant cellulose nanofibre (CNF) isolation from lignocellulose biomass.

According to Madsen [51], the plant primary cell wall consists of 9–25% cellulose microfibrils, compared to the secondary wall that contains 40–80%. Furthermore, the primary cell wall consists of 25–50% and 10–35% of hemicelluloses and pectin, while the secondary cell is composed of only 10–40% hemicelluloses and 5–25% lignin. However, the precursor material referred to as lignocellulose biomass should be cleaned and dried. After the isolation of lignocellulose biomass, it still contains many accompanying materials apart from cellulose such as hemicellulose and lignin. Figure 4 presents the structure of a typical plant cell, presenting details of the layers and the accompanying materials that the cell wall is composed of. The removal or elimination of accompanying materials is required as hemicellulose carries carboxylic groups, which give the final CNFs a slightly negative charge, causing repulsion between the fibres and preventing their aggregation in the wet state [52]. However, the isolation of CNFs can be conducted in two main steps, which are the pre-treatment and production steps. The first step involves the pre-treatment of cellulose fibres (CF) while the second step involves the production of cellulose nanofibre (CNF), as presented in Figure 5 [50]. To isolate nano cellulose (NC) from lignocellulosic biomass, it must undergo delignification, which is a necessary process consisting of pulping until depolymerisation, followed by the solubilisation of lignin and hemicelluloses, then by bleaching with chemical agents [50,53].

Native cellulosic fibres must be treated in strongly acidic conditions to hydrolyse the amorphous fractions of cellulose and form simple, highly crystalline rod-like cellulose nanowhiskers. These whiskers have been termed differently in many works of literature as nanowhiskers, cellulose nanofibres, cellulose nanofibrils, micro fibrillated cellulose or nanofibrillated cellulose (NFC). Figure 5 shows the steps of cellulose nanofibre (CNF) isolation from lignocellulose biomass.

NFC can be isolated from biomass at high shear forces with high-pressure homogenisers. The results from previous studies revealed that enzyme hydrolysis has the potential to enhance the surface charge and the mechanical activation of the degree of freedom of carboxymethyl of the isolated CNF. The modification of nanofibrils can be achieved by esterification of the primary and secondary hydroxyl group present in NFC by mechanical disintegration [55].

According to Wang et al. [56], CNF aerogel can be prepared from pure CNFs by two main steps: the freezing of poly(aminoamide) epichlorohydrin resin with CNF mixture in liquid nitrogen or the freeze-drying of the frozen mixture. The CNF aerogels will be obtained after freeze-drying at −80 °C, 15 Pa, for about 72 h. However, other techniques for the fabrication of CNF aerogel have also been developed apart from freeze-drying. Toivonen et al. [57] developed a method of cellulose nanofibre vacuum filtration, then they exchanged the solvent and ambient drying that produced aerogel membranes. They developed this novel approach to avoid critical point drying (freeze-drying). Drying approaches such as freeze-drying (FD), supercritical drying (SCD), spray drying (SD), and oven drying (OD) are still the most widely used techniques for the preparation of nano cellulosic particles. The comparison in Table 2 presents the various drying techniques use in the production of CNF aerogel.

### 3.2. Isolation of CNF Aerogel

**Figure 6:**
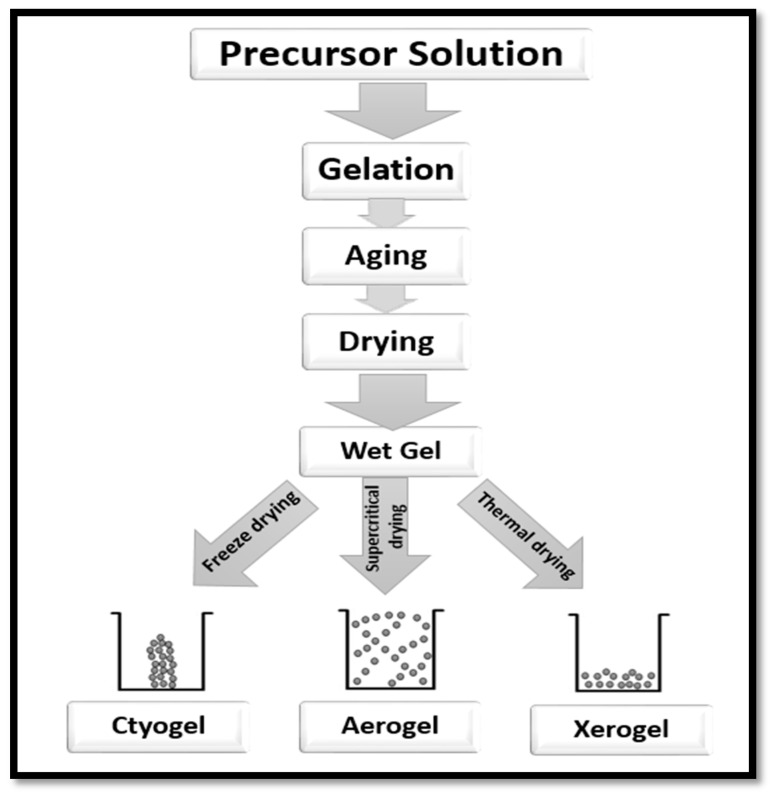
Steps of preparation of aerogel from a precursor solution.

Aerogel is a primary component of a solid network of loosely bonded fibres or particles of precursor materials. It determines the density, porosity and other properties of the network. It is known to be lightweight, to have open pores, extremely small feature sizes and is endowed with a significantly high specific surface area (SSA) [61]. The preparation of aerogel involves three main steps. The first step is the sol–gel transition, often referred to as gelation; the next step is network perfection (ageing), and the final step is the period of gel–aerogel transition, which is known as the drying stage (Figure 6). The precursor material is first dispersed in a proper liquid to form a colloid and then left in order to enable gelation [15]. The colloid solution forms the gel through a strong interaction with the added cross-linking agents [62].

Numerous factors influence the gelation time of the colloidal solution. The precursor material, concentration of the colloid solution, the additives (if any) and the parameters of the solutions such as pH, temperature, etc., [62,63,64]. After the gelation time, many precursor materials require some additional steps to strengthen the gel network [62]. Drying of the gel network takes place after the completion of gelation time. The surface tension of the pores of the solid network is minimised with freeze-drying to form cryogel, or thermal drying to form xerogel or supercritical drying process to form aerogel (Figure 6).

### 3.3. Properties of Aerogel and CNF-Based Aerogels

#### 3.3.1. Physical Properties and Surface Area

Aerogel properties differ based on the precursor material, methods of preparation and the additives incorporated in the material. However, nanocellulose-based aerogels are ultra-light and highly porous, with an extremely low density [65,66]. Hence, for aerogels with a porosity higher than 99%, the bulk density is inversely proportional to the porosity and directly proportional to the initial concentration of the precursor material. Tuukka et al. [61] obtained CNF aerogels of different concentrations of CNFs impregnated with bio-based epoxy via vacuum infusion and confirmed that the initial concentration of CNF was directly proportional to the density and inversely proportional to the porosity and Brunauer–Emmett–Teller (BET) specific surface area. The porosity of aerogel results from a fibre network that has been dried. The more fibre in the network, the less pore size and porosity. Aerogels generally exhibit lower compressive strengths and decreased elastic moduli when they are reinforced with fibres. This is due to the greater matrix densities observed for unreinforced and slightly reinforced aerogels relative to highly reinforced aerogels. However, nanofibre has a low density, unlike silica aerogel, because of its optical transparency, and a low surface area without linear expansivity. The density of nanocellulose aerogel can be reduced by adding more octylamine [65]. During the solvent exchange in the preparation of nanocellulose aerogel, the volumetric shrinkage can be reduced by modifying the concentration of initial nanocellulose particles [67,68], the formation of a percolative network, or making a cross-link between the nanocellulose particles [68]. A porous structure can be obtained with this method. Furthermore, minimised shrinkage can also be achieved by adjusting the conditions of the drying process to a −20 °C temperature (freezing), after which supercritical CO_2_ is released [69]. The drying process, convection and high moisture should be removed to minimise drying stress, which may lead to the collapse or shrinkage of the aerogel [65]. The high specific surface area (SSA) of aerogels is very desirable for most of their applications. The SSA for aerogels can be significantly increased by avoiding pore closure in the aerogel, which can be accomplished by changing the solvent from water to butanol [70].

#### 3.3.2. Mechanical and Morphological Properties

The mechanical properties of cellulose nanofibre aerogel are closely dependent and explained based on the morphology of the aerogel. The larger the pore sizes in the aerogel 3D network, the lower the mechanical strength of the aerogel. Furthermore, the pore sizes of a fabricated aerogel are majorly dependent on the initial concentration of the precursor material and the method of preparation of the aerogel. However, the morphology of aerogels is typically influenced by many factors which include the precursor material, preparation method, additive materials, cooling rate, and physical conditions. According to Sakai et al. [69], a supercritical drying approach for CNF-based aerogels can create small and open pores in the aerogel. To create small pores with more homogeneity in their structure, a rapid cooling rate should be applied to the aerogel [71]. Increasing the initial particle concentration will lead to an increase in aerogel density, as mentioned earlier, primarily caused by the decreasing pore size with a more regular structure [72,73]. Finally, another factor affecting the porosity of aerogels is that additives such as fillers or any other materials may alter the architecture of the aerogel’s porosity [74]. According to Svagan et al. [75], starch added to nanocellulose based aerogel (NBA) could lead to a reduction in the size of the pores by approximately 65%. Depending on the material they are prepared from, aerogels tend to be sensitive to moisture absorption from handling and storage, are more brittle with ageing and may exhibit stress relaxation (or creep) under certain conditions. KE Parmenter et al. [76] tested the tensile and shear properties of silica aerogel and suggested that the aerogels have low tensile strengths relative to compressive and shear strengths, which is typical of brittle materials. Apart from production costs, CNFs can be categorised as one of the most superior reinforcing materials for biopolymers because of their inherent potential, such as their biocompatibility, renewability, biodegradability, and universality. CNFs can be isolated from various sources, as mentioned earlier. The unique structure of CNFs makes them possess excellent mechanical and morphological properties and makes surface modification easy [77,78]. The properties of CNFs differ depending on the source of cellulose and the preparation methods that are used in the isolation process [78]. Compared to traditional cellulose-based materials in aerogel production, CNFs have a low shrinkage and a higher crystallinity, resulting in highly porous aerogel [12]. Recently, many reinforced fibres, supporting materials, and even other polymers have been integrated with CNFs for aerogel production, resulting in a diverse novel material with different mechanical properties. Moreover, during the solvent exchange of aerogel preparation, CNF is prone to self-agglomeration and this slows down the advancement process of CNFs in aerogel production [55]. Cellulose nanofibres can be functionalised in their natural state without altering their original molecular structure, and this explains their dispersion in polymeric composites. Furthermore, the increase in mechanical strength when nanomaterials (for example, CNFs) are used for reinforcement is majorly attributed to the increase in the surface area of interaction with the polymer matrix. CNFs, based on their intrinsic biodegradable properties, have been used as reinforcements for biopolymers in high-strength applications [79].

#### 3.3.3. Biocompatibility and Toxicity

The selection of materials is one of the most important properties of any proposed material for biomedical or biological applications. The essential characteristics are defined by its biocompatibility and toxicity level. Biocompatibility is a major test used in the biomedical application, which is done either by an in vivo or in vitro method. The functionality of the biomaterial determines its appropriateness for specific medical applications and its ability to initiate a host response. Several biopolymers have been tested for their compatibility with a biological system using different lab animals such as rats [80]. Fernández-Cossío et al. [81] are one of notable researchers in the study of the bioactivity and biocompatibility of cellulosic materials. Agarose gel was investigated for usage in a subcutaneous implant [70].

The most commonly applied assay for evaluating biocompatibility is in vitro cytotoxicity analysis, which measures the effects of the material on cells after exposing them to the material and is usually performed in less than 96 h [82]. Precursor materials play a critical role in the biocompatibility and toxicity of the aerogel. Other contributing factors are the chemicals used in the preparation of cellulose, cellulose nanofibre and cellulose nanofibre aerogel. Table 3 shows the summary of selected studies that used different preparation techniques. Extracted CNF using enzyme hydrolysis has been reported with no cytotoxicity at any concentration [83]. Similarly, the cytotoxicity of isolated cotton cellulose nanofibres with bovine fibroblast cells by the acid hydrolysis method showed that the toxicity of CNF depends on its concentration [84]. During the isolation of CNF, it is exposed to some physical, chemical, and even mechanical changes that may affect its toxicity to cells. Many studies confirm that there is no sign of toxicity in pure CNF [85,86,87], and other studies reported low or no significant toxicity [84,88], making it possible that either the preparation method or the source of CNF affect the cytotoxicity level. The long-term effect of most of the used materials is yet to be revealed, as well as the impact of them inside the body. The nanoparticles of many polymers can induce a subsidiary effect on cells, which may take a long time and is not possible to observe in a few days. Bacterial cellulose has been reported as innocuous, with no signs of any cytotoxicity in the subcutaneous tissue of mice, and this material has been proven for use in the production of tissue engineering grafts [89]

Many studies have suggested, after different tissue cultures, that CNFs showed good biocompatibility, and no cytotoxicity was observed. Table 3 presents a summary of some of these studies. Most of the toxicity studies presented were done on the basis of cytotoxicity by using cell tissue cultures, which may not give an exact image of the compatibility of the selected materials. However, CNFs and CNF-based aerogels, when compared to other materials, are still preferable due to their low cytotoxicity.

Miyamoto et al. applied in vivo methods for appraising the absorbance by living tissue and foreign body reaction for cellulose and some derivatives including methylcellulose, ethylcellulose, hydroxyethylcellulose, aminoethyl cellulose, and cellulosic polyion complexes. It was observed that absorbance by living tissues depended on the crystallinity and chemical structure of polymers, while foreign body reaction was mild and almost the same for all the tested samples, showing that cellulose could be considered as a biocompatible material by some physical and/or chemical transformations [92]. The synthesis of microbial cellulose by *Acetobacter xylinum* was effective for wound healing applications. The study of Czaja et al. [93] revealed that the physical properties of microbial cellulose are determined by the genetic modification of the host organism; this was attributed to the nanofibrillar structure of microbial cellulose which had the propensity to exhibit a perfect matrix as an optimal wound healing environment. As a natural material, cellulose nanofibre-based aerogels have the potential to be involved in many future medical applications, even though there have been some concerns about using nanoparticles; however, more long term research needs to be done to analyse and evaluate the possibility of any long term effects of them on the human body.

## 4. Cellulose Nanofibre (CNF) Aerogels in Medical Application

Cellulose nanofibres (CNFs) are usually converted to aerogel during drug absorption. The properties of CNF aerogels such as their biodegradability, biocompatibility, low toxicity and renewability have had an impact on the prospects of aerogels in a wide range of biological and medical applications under investigation (Table 4). CNF aerogel has been tested in diverse medical applications such as biosensing, tissue engineering [94] and drug delivery [95].

### 4.1. Tissue Engineering

In tissue engineering technologies, to maintain regular growth of tissue, the cells must have a 3D scaffold to permit a proper exchange of waste or nutrients for the growing cells [106]. CNF aerogels have been used as a scaffold for tissue engineering and are proven to enhance the growth and the proliferation of cells [105,113,114]. The high porosity (more than 99%) of CNF-based aerogels allows high oxygen permeability and accelerates the exchange of metabolic requirements in growing cells, leading to enhanced cell activities, better adhesion, and increased proliferation. Unlike the other scaffolds, CNF and bio-based scaffolds have less cytotoxicity to growing cells, and their biocompatibility has proved to be higher. Liu et al. [113] reported that CNF and bio-based scaffolds had less than 5% cell death after 72 h of cell growth.

**Figure 7:**
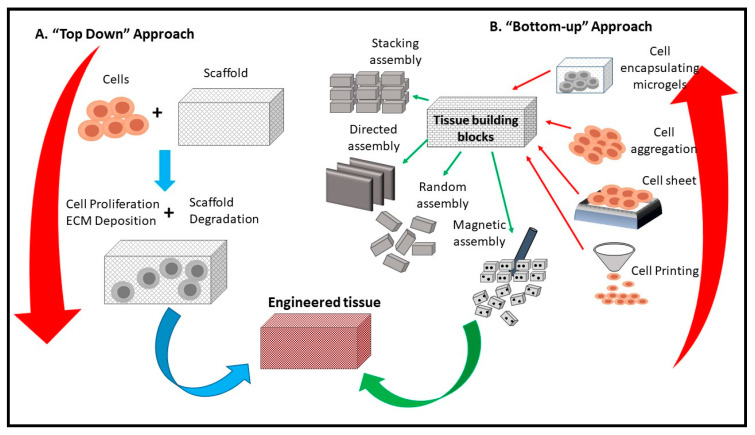
Schematic diagram of the use of CNF in tissue engineering (adapted from Tingli Lu et al. [115]. Copyright 2013. Reproduced with permission from the Dove Medical Press publisher.

Similarly, Cai et al. concluded that CNF aerogel microspheres significantly facilitated the growth and proliferation of fibroblasts [106]. Scaffolding is an essential part of tissue engineering, besides giving the oxygen, adhesion and metabolic requirements to cells, it also directs the shape and structure of the tissue. Figure 7 presents a schematic diagram of a CNF-based scaffold and its usage in tissue engineering. Scaffolds can be designed based on the needed tissue, thus allowing the target cells to proliferate accordingly. Numerous composite aerogels have been prepared recently, containing different materials; Lu et al. [105] prepared composite aerogels using cellulose and collagen as precursors to reduces the influence of protease, thereby curing chronic wounds. This is because collagen is biocompatible, biodegradable and has strong adhesive characteristics on the skin. Hence, it is applicable for wound dressing.

#### 4.1.1. Wound Healing

**Figure 8:**
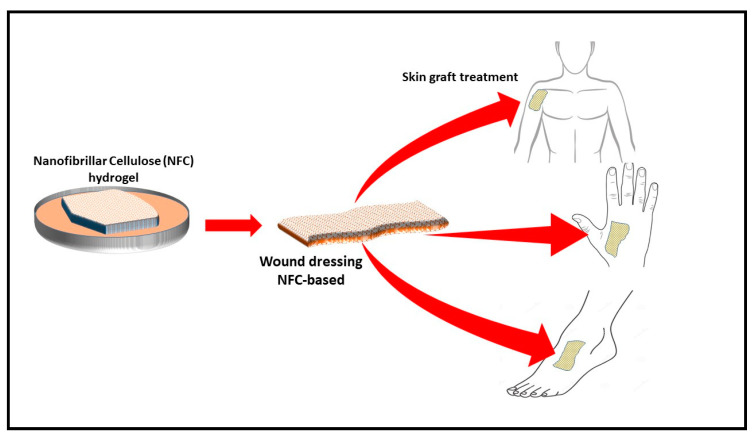
Schematic drawing of production of biodegradable antimicrobial wound dressing material (adapted from T. Hakkarainen [119] et al., 2016). Copyright 2016. Reproduced with permission from Elsevier Ltd.

The process of wound healing requires normal cellular function, moisture control, and optimal oxygen permeability [56,88,116]. The effective surface area of filamentous biomaterials has been explored to develop nanoparticle-based cellulose. For example, a nanocellulose polymer was developed for cellular uptake. Similarly, a synthesised polymer was used as a folate receptor for the testing of cancer [117]. However, CNF aerogel can be incorporated with an antimicrobial material during the preparation process and can be used as a wound dressing material (Figure 8). Many plant extracts have been investigated for their antimicrobial activity. An evaluation of the effect of aqueous and alcoholic extracts of the peels of Punica granatum showed better activity than some of the common antibiotics. Similar materials can be incorporated in the aerogel to generate an antimicrobial aerogel for wound dressing [118]

Antimicrobial-incorporated CNF aerogel prevents infection and provides moisture and optimal oxygen permeability to the cells for proliferation. CNF aerogel has an advantage in that regular wound dressing sheets may not provide oxygen permeability or microbial control. The use of cellulose nanofibre aerogels with their high porosity and surface area provide oxygen permeability, thereby preventing the growth of anaerobic bacteria. Moreover, they can be incorporated with an antibacterial ingredient to inhibit aerobic bacterial as well. Wang et al. [88] investigated CNF and copper-containing mesoporous bioactive glass as antibacterial ingredients in aerogel composites for the potential treatment of chronic wounds. The obtained aerogel completely inhibited the growth of *E. coli* and other inflammatory bacteria. However, using metal particles like copper and sulphur is not desirable for many researchers, due to the toxicity potential. CNF incorporated with natural antimicrobial material aerogel (CNF/AM aerogel) could serve as a potential treatment for chronic diabetic ulcers. CNF/AM aerogel provides the sustainable inhibition of the microbial growth on wounds. The high porosity of aerogel provides aerobic conditions, preventing the growth of anaerobic bacteria such as *Clostridium perfringens* and gangrene development. Moreover, antimicrobials incorporated with the aerogel could prevent the growth of infectious aerobic bacteria and accelerate wound healing.

#### 4.1.2. Biosensing and Diagnostics

A biosensor is known as an analytical device that detects low concentrations of a desirable parameter, and it combines a biological component with a physicochemical detector. Nowadays, with the development of nanotechnology, the use of 3D frame materials like bioaerogels is a crucial strategy to overcome the challenges of the low instability and sensitivity associated with 1D and 2D materials. Low-density nanocellulose aerogel from cotton has been applied as a transducer biosensor surface for protease wound dressing (Edward et al., 2016). Bacterial CNFs incorporated with gold nanoparticles have been used to immobilise heme proteins and enzymes due to the extensive surface area and biocompatibility of nanofibres. This matrix of bacterial CNF incorporated with gold nanoparticles was used to fabricate hydrogen peroxide biosensors. The findings revealed that they have the capability of detecting low concentrations of hydrogen peroxide [66].

**Figure 9:**
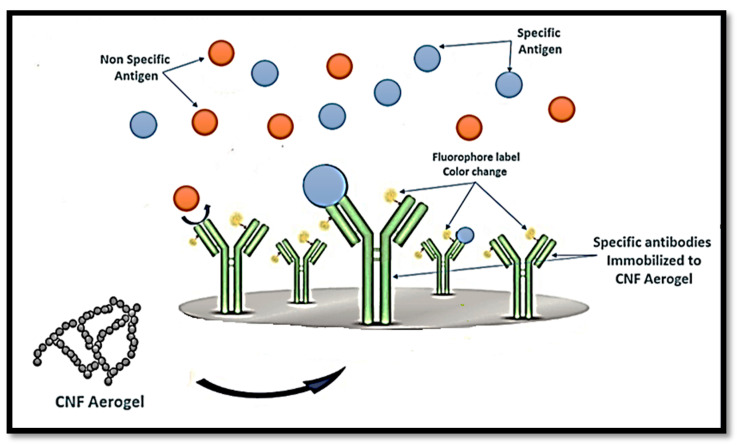
Schematic diagram of the potential use of CNF aerogel in the diagnosis of specific antigens.

Similarly, Weishaupt et al. [120] developed a biosensor for heavy metals. The sensing of cyanobacterial biomolecule C-phycocyanin (CPC) was enhanced with genetic modified bacteria and integrated into CNF films as a carrier material, after which CPC–CNF films were able to detect free copper ions in human blood serum, and heavy metal sensitive fluorescent emission was observed. CNFs in aerogels can also be linked to either immobilised antigen or antibodies that conjugate to an enzyme or fluorophore label used to detect the specific antibody or antigen, respectively, as presented in Figure 9.

#### 4.1.3. Drug Delivery

In recent decades, wide varieties of novel delivery systems were developed and reported on in relation to the prolonged and controlled release of pharmaceuticals and other bioactive compounds through different routes of administration. Hydrophobic nanocellulose aerogels are one of these controlled systems, and can increase drug bioavailability compared to the intravenous and oral bioavailability of the pure drug solution. Moreover, the other advantages include the mucoadhesive properties and the floating tendency of cellulose bioaerogel [103]. The use of CNFs has been explored in drug formulations due to their unique physicochemical properties. These unique properties include their rheological and barrier properties. These properties are responsible for the stability of CNFs in oil–water and air–water interfaces. This unique characteristic can also be attributed to their large surface area to volume ratio, which means that molecular interactions will require less soluble drugs. However, by varying the porous structure of CNF aerogels, and controlling the interactions between them and the desired drug to be delivered, the drug release profile can be tuned [104,121].

**Figure 10:**
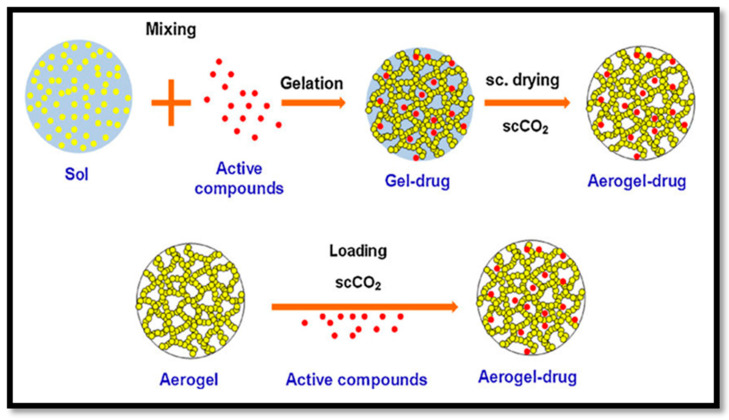
Fabrication process of drug delivery aerogel (cited from García-González et al. [122]. Copyright 2011. Reproduced with permission from Elsevier Ltd.

Paclitaxel is an anticancer drug that has been effectively delivered to human-derived tumours in a mouse model with aerogels. A wide variety of other drugs were recently delivered in animal models following the same approaches, as illustrated in Figure 10. The drug delivery system can be controlled by controlling the size of the pores, surface area and drug and aerogel interactions. This approach could be a novel treatment for type one diabetes. Beta cell (β cell) pancreatic islets found in the pancreas that synthesise and secrete insulin could be immobilised in CNF aerogel and masked from our bodies’ immune systems. These aerogel-immobilised β cells could be introduced to the human body as a potential biosynthetic pancreas.

#### 4.1.4. Antimicrobial Immobilisation

Cellulose nanofibre incorporated with natural or synthetic antimicrobial material can be immobilised inside the nanocellulose fibres (NCFs) based aerogels. This complex can be fabricated and then applied as an antimicrobial film for wound dressing. Many studies have been carried out regarding the incorporation of CNFs with silver and zinc as antibacterial inhibitors acting on different targets inside the microbial cell. Uymin et al. [110] have incorporated lysozymes and silver nanoparticles on the surface of CNF aerogels, resulting in the inhibition of the growth of 99.9% of all tested microbes. Table 5 summarises some examples of the immobilisation of antimicrobial materials inside the NCFs. In the study conducted by Xiao et al. [123], the authors investigated the efficacy of antibacterial nanocellulose-based sponges through the covalent immobilisation of gentamicin. The result obtained revealed that the incorporation of 0.33 wt% gentamicin in the CNF sponge has a non-leaching effect and a durable contact-active antibacterial effect. Although there was no significant effect on the surface morphology, the findings revealed that cross-linkages slightly influenced the mechanical properties.

### 4.2. Potential and Challenges of with CNF Aerogel in Biomedical Application

The potential use of CNF-based aerogels as sustainable materials for biomedical applications is cost effective. However, thorough toxicity testing of all nano-biomaterials remains essential, yet few studies have been reported in the literature. Specifically, detailed in vivo and biocompatibility studies on the interaction between CNFs and biological tissue have not been unexplored. The available literature failed to compare the CNFs’ sources with the types of animal models used. Many studies have been reported regarding the use of CNF-based materials for different applications. Nevertheless, CNFs still have some drawbacks and challenges that may restrict their usage in biomedical applications due to their high production costs. This is because most of the production routes use supercritical drying, which is expensive and risky. This shows that investigations into CNF production on the laboratory scale are yet to be considered for wide-scale commercialisation purposes. Microstructural images of some of the previously produced CNF-based aerogels are presented in Table 6. Their morphological characteristics, as illustrated by the SEM images, show a good porous nested network, which promotes the growth of damaged tissue when they are used as implants.

However, at present, the main challenge of CNFs is related to the development of a green isolation process of nanocellulose from the natural cellulosic biomass. The current utilisation of harsh chemicals increases the toxicity of the isolated CNF. The preparation of bacterial celluloses (BCs) via the use of enzymes make the process greener and mostly acceptable in the laboratory as well as on an industrial scale. On the contrary, they have a high yield of production and less energy consumption. They are also less costly compared to chemical processes, and they also avoid pollution. Therefore, the utilisation of the enzymatic approach for the production and further modification of nanocellulose has lots of advantages compared to the adoption of the chemical approach.

Many studies have been conducted on the creation of a mass synthesis process for bacterial cellulose nanofibre, which significantly reduces the production cost. However, large-scale industrial production of bacterial cellulose nanofibre has not been achieved. Furthermore, more biochemical and genetic studies are needed to fully comprehend and enhance the cellulose production process. Another challenge is the poor processing potential of cellulose nanofibres due to their insolubility in most solvents. The poor solubility behaviour of cellulose nanofibres is attributed to their high crystallinity. Studies have been conducted to enhance the solubility of the cellulose nanofibres with the use of ionic liquids. The crystal structure of the cellulose is often destroyed with the solubility enhancement procedures and this results in poor mechanical properties in the cellulose. Despite extensive advancements in tissue engineering, no materials have been found to capture the intricacies of the native tissue nor its functionality to an ideal level. The challenge of innovating new composite materials with nanoscale engineering methods to produce fully biomimetic tissues has not yet been achieved.

According to the Cryogel Safety Data Sheet (2007), from a health perspective, aerogel may irritate the eyes and skin of some people. Furthermore, the nanosized particles of aerogels can cause silicosis in the respiratory tract if inhaled. The particles may also cause dryness in the eyes, mucous membranes and even skin. Other challenges such as CNFs’ toxicity, their environmental impact and the high-energy consumption of their production route need to be researched in detail. Furthermore, the biological and environmental toxicity of CNF-based materials’ remediation may be the greatest obstacle for their application and marketability, as the eco-toxicology studies on CNF-based materials are still limited and are at the primary stage [136]. However, more research needs to be performed to confirm whether CNF-based materials have any toxicity to humans, animals, or even microorganisms.

## 5. Conclusions

Plant cellulosic nanofibres (CNFs) have attracted the interest of scientists for use in the production of sustainable biomedical materials. The effective design of plant CNF-incorporated aerogel materials can be a useful tool for the development of sustainable anti-infection materials for wound dressing, scaffolds for tissue culture and drug delivery systems. The primary role of their design is defined based on the possibilities, limitations, and suitability of CNF aerogels for the development of sustainable material. Previous researchers have achieved significant progress; however, in the literature, there have been reports of challenges in the commercialisation of sustainable biomedical materials based on CNF aerogels. It has been reported that further lab-scale toxicity experiments and animal model studies on the use of CNF aerogels as sustainable materials in biomedical applications are necessary.

## Figures and Tables

**Table 1:** Summary of the evolution of aerogels.

Year	Types of Aerogel	Precursor Material	Preparation Method	References
1931	Silica aerogel	Sodium silicate	Supercritical drying	[3]
1932	Organic and metal oxide aerogels	-Metal oxides -Organic compounds	Supercritical drying	[14]
1950	Hydrophobic silica aerogels	Single and combinations of metal oxides with silica	Silylation with trichloro-methyl silane to produce water repellents	[16]
1968	Sol–gel route silica aerogel	Single and combinations of metal oxides with silica	Sol–gel route replaced water glass with TMOS then removed at supercritical conditions	[16]
1974	Sol–gel route silica aerogel	Single and combinations of metal oxides with silica	Sol–gel route silica aerogel	[18]
1989	Organic and carbon aerogels	Organic polymer	Sol–gel route silica aerogel	[19]
1992	Low-density and high-porosity silica aerogel	Single and combinations of metal oxides with silica	Acid–base process and substitution of the alcohol with an aprotic solvent	[17]
1996	Silica aerogel	-Metal oxide -Organic polymers	Developing rapid supercritical extraction (RSCE)	[20]
1997	Ultralight aerogels or X-aerogels	-Metals -Organic polymers	Crosslinking di-isocyanates into the silica structures inside the aerogels.	[21]
2006	Cellulose-based aerogels	Cellulose derivatives	Nontoxic iso-cyanate, via the sole gel route, with a tin-based catalyst.	[22]
2008	Cellulose nanofibres (CNF) aerogel	Cellulose nanofibres (CNF)	Facile vacuum drying of aqueous CNF gel.	[23]
2009	Metal aerogel	-Metals -Organic polymers	Smelting interpenetrating of resorcinol-formaldehyde and iron oxide xerogels	[24]
2012	Cellulose nanowhisker foams	Fully bleached commercial softwood Kraft pulp	Prepared via a freeze-casting method	[25]
2014	Aerogel-based Plasters	Silica + natural plaster	Granular silica aerogel mixed with natural plaster in different percentages	[26]
2015	CNF aerogel with water absorbency and shape recovery	Cellulose nanofibrils (CNF)	Crosslinking of CNF by the reaction between the C–C double bond of maleic acid-functionalised CNF and hypophosphite.	[27]
2016	Superhydrophobic and ultralight cellulose-based aerogel for oil spill	Cellulose-based aerogel	Novel physical-chemical foaming method, plasma treatment, and subsequent silane modification process.	[28]
2017	NFC Aerogel with thermal super-insulating	Nanofibrillated cellulose (NFC)	Spray freeze-drying (SFD) of Cellulose nanofibrils	[29]
2018	Low-cost method of silica aerogel	Fly ash and trona ore	Ambient pressure drying technique	[30]
2020	Agricultural Bio-waste as a Novel Cellulose Aerogel	Tea stem wastes (TSW)	Pure raw cellulose was isolated, hydrogel formation and then freeze-drying to form cellulose aerogel.	[31]
2020	A novel multifunctional carbon aerogel	β-TCP powders and sodium carbonate, formaldehyde	Beta-tricalcium phosphate bioceramic was platform-coated with carbon aerogel.	[32]

**Table 2:** Comparison of various nanofibrillated cellulose (NFC) drying approaches [58,59,60].

Name	Method	Particle Size	Advantages	Disadvantages
Freeze-drying	Freezing of CNF suspension at −65 ℃ then lyophilisation	μm to mm and nanosize thickness	One well-established nanodimension	Expensive agglomeration
Supercritical drying	Dehydrating the NFC suspension and replacing the solvent with L(CO_2_)	Nanosize	Dimensions stay in nanosize	Expensive and complicated method.
Spray drying	Concentrating and pumping the liquid then, dehydrating by hot gas	7.48 μm	Controllable size and not expensive	Agglomeration
Oven drying	Put the suspension of NFC inside the oven at 105 ℃ for 24 h.	>100 μm or even mm	Well established for the industry	Loose of nano-D, Bulk material generation

**Table 3:** Toxicological evaluation of CNF-based materials.

Material	Preparation Method	Toxicological Experiment	Conclusion	Reference
Micro-fibrillated cellulose (MFC)	Fibrillating the fibres under high compression and shear forces.	Cytotoxicity evaluation with mouse macrophage and human monocyte	No evidence of cytotoxicity from the material nor the method.	[85]
Cotton cellulose nanofibres	Acid hydrolysis method	Cytotoxicity evaluation bovine fibroblast cells In-vitro effect on gene expression	Low cytotoxicity at low CNF concentration Reduction in cell viability and affection of expression of stress and apoptosis AMM at high concentration	[84]
Poly(vinyl alcohol)/cellulose nanofibril hybrid aerogel	Emulsification and freeze-drying processes	Cytotoxicity investigated with NIH 3T3 cells to explore their potential application as cell culture scaffolds.	Aerogel facilitates cell attachment, differentiation, and proliferation. Moreover, it was nontoxic and biocompatible	[90]
Cellulose nanofibril-based structures	Homogeniser without pre-treatment and with 2,2,6,6 tetramethylpiperidine-1-oxy radical	Cytotoxicity evaluation with 3T3 fibroblast cells	No toxic phenomena for pure CNF and slight toxicity for modified CNF	[86]
Cellulose nanofibres	Mechanical grinder preceded by mild chemical treatment	Cytotoxicity assays using a Vero cell lineage.	No cytotoxic behaviour of CNF or the method for direct and indirect assays	[87]
Cu/mesoporous bioactive glass/CNF membranes and aerogels	EISA method for MBGs, freeze-drying for membrane and solvent-exchange-freeze-drying for aerogel.	Cytotoxicity and biocompatibility evaluation in a 3T3 mouse fibroblast	Low cytotoxicity at low modified CNF concentration and no cell growth in high concentration	[88]
Cellulose nanofibres	Enzymatic hydrolysis method	The cytotoxicity of CNF assessed by MTT assay against three different cancer cell lines NCIH460, PA1, and L132 cells.	CNF did not show the cytotoxic effect at the tested concentrations in any of the cell lines.	[83]
Resveratrol-loaded cellulose aerogel	Freeze-drying method	Cytotoxicity to cartilage cells by the standard MTT assay	Low toxicity and good biocompatibility.	[91]

**Table 4:** Chronological examples of some studies with cellulose and CNF-based materials in biomedical applications.

Material	Advantage	Method	Application	Reference
Super critically dried silica sol-gel discs	Facilitate the detection of chemicals and organisms	Use of viruses to trigger a response in immobilised bacteria and chemicals	Biosensors and diagnostics	[96]
Cellulose-based hydrogel	Superabsorbent capacity and satisfying biodegradability	Tested for biodegradability and antibacterial activity against E.coli	Antibacterial activity	[97]
Ultrafine cellulose acetate fibres with silver nanoparticles	Very strong antimicrobial activity	Direct electrospinning of a CA solution with small amounts of AgNO_3_ and then photoreduction	Antimicrobial film	[98]
Cellulose acetate nanofibre	Inhibit the growth of G+ and G- bacteria	cellulose acetate nanofibre membrane using supercritical carbon dioxide	Strong antibacterial film	[99]
Hydroxyapatite/bacterial cellulose (HAp/BC) nanocomposite	Better adhesion and activity and faster proliferated	HAp/BC nanocomposite scaffolds were prepared to utilise the biomimetic technique	Bone tissue engineering.	[100]
Bacterial cellulose (BC) aerogel	Easily equipped No aide interactions	BC aerogel matrix loaded with drug and the release behaviour from the matrix were studied	Drug delivery	[101]
Bacterial CNF incorporated with gold nanoparticles	Biocatalytic activity and fast response in low conc. of H_2_O_2_	Immobilisation of heme proteins and enzymes	Fabrication of H_2_O_2_ biosensors.	[102]
Hydrophobic nanocellulose aerogels	Increase oral availability of drugs	Physical adsorption of a drug to aerogel for oral administration	Drug delivery system	[103]
Nanofibrillated cellulose (NFC) aerogels	Controlled drug delivery	NFC hydrogels are incorporated with the drug then convert it to aerogel	Drug delivery system	[104]
NCF/collagen composite aerogels	Strong absorption Biocompatible High proliferation.	Di-aldehyde NCFs and collagen were cross-linked together and formed the composite aerogels.	Tissue engineering and wound dressing	[105]
Nanocellulose aerogel (NCA)	Significant increase in cell count.	Cultured NIH 3T3 cells for two weeks on NCA.	Scaffolds for 3D cell culture	[106]
Nanocellulose aerogel (NA)	Monitor the level of protease in chronic wounds	The complex of polypeptide-NA (PepNA) to detect the sensitivity of PepNA for human neutrophil.	Biosensors	[107]
Antibacterial cellulose-based aerogel	Bacterial inhibition rate of >99.99%.	Fixing antibacterial substances on the surface of cellulose aerogels.	Bacterial growth inhibition	[108]
CNF composite aerogel	Significant increase in cell count.	Cultured 3T3 NIH cells on poly (vinyl alcohol).	Scaffolds for 3D cell culture	[90]
NFC aerogel	Noticeable increase in drug release	Loaded of NFC aerogel with alkylating antineoplastic agent.	Cancer treatments	[109]
Nanocellulose derivate aerogel	Complete inhibition of tested bacteria.	Loading lysozymes and silver nanoparticles on CNF aerogel.	Bacterial growth inhibition	[110]
Strain-sensing protonated CNF aerogel	Stretchable and sensitive	Cross-linking CNF surface with PSS in PEDOT/PSS generated PEDOT/PSS/CNF aerogels	Biosensors	[111]
Nanocellulose/gelatine composite cryogels	Controllable porosity, and good biocompatibility	Used cross-linked di-aldehyde starch as carriers for controlled 5-fluorouracil (5-FU) release.	Controlled drug release	[112]

**Table 5:** Immobilisation of antimicrobial ingredients into CNFs.

Antimicrobial Agent	Function	Reference
Silver nanoparticles (average size of 21 nm) incorporated into the cellulose acetate nanofibre	Excellent antibacterial action against Gram-positive *S. aureus* and Gram-negative *E. coli, K. pneumonia,* and *P. aeruginosa*	[124]
Silver nitrate (size ranging from 10 to 20 nm) incorporated into the cellulose acetate nanofibre	Very strong antimicrobial activity against *S. aureus, K. pneumonia, E. coli,* and *P. aeruginosa*	[125]
Composition of nanofibrillated Cellulose with silver nanoclusters (NFC/AgNC)	Antibacterial activity against *E. coli*	[126]
ZnO incorporated into the cellulose acetate nanofibre	Exhibited strong antibacterial activity against *S. aureus, E. coli,* and *Citrobacter*	[127]
Silver nanoparticles incorporated into bacterial cellulose nanofibres	Strong antimicrobial potential against *E. coli* and *S. aureus* bacteria	[68]
T4 bacteriophage incorporated into core/shell electrospun fibres of polyethene oxide, cellulose diacetate (CDA) and their blends	Prevent bacterial growth on contaminated food surfaces	[128]
Porous CNFs with biomass tar, polyacrylonitrile (PAN), and silver nanoparticles	Excellent antimicrobial performance against Gram-positive *S. aureus* and Gram-negative *E. coli*,	[129]
Chitosan adsorbed cellulose nanofibre (CNF) films	Prepared CNF film even with low Mw of chitosan exhibited antibacterial activity against *L. innocua* and *E. coli.*	[130]
Covalent grafting of gentamicin to nanocellulose-based sponge	Excellent antibacterial performance against *E. coli* and *S. aureus*, with bactericidal rates of over 99.9%	[123]
Ag nanoparticle/cellulose nanofibre (Ag NP/CNF) composite aerogels	The aerogel exhibited good antibacterial (for *E. coli*) and antifungal (for *A. niger*) activity.	[131]
Cellulose nanofibres (CNFs) and thyme essential oil (EO)	Sustained antibacterial release for fresh food preservation.	[132]

**Table 6:** SEM images of cellulose nanofibrillate aerogel. Reproduced with permission from Springer Nature © 2018, Scrivener © 2017, and the Royal Society of Chemistry © 2013.

SEM Images of CNF-Based Aerogels		Type	Reference
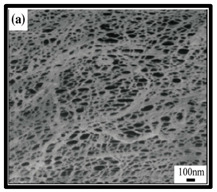	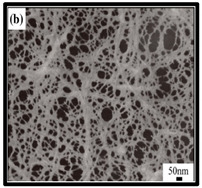	(a) and (b) pure CNF aerogels.	[133]
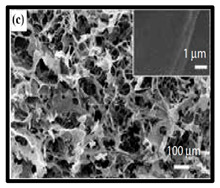	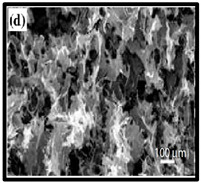	(c) neat CNF, (d) CNF/ carbon nanotubes (CNTs) (75/25 wt%).	[134]
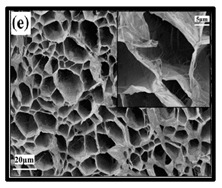	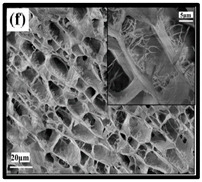	(e) uncoated Polyvinyl alcohol (PVA)/CNF aerogel, (f) coated PVA/CNF aerogel.	[135]
